# Supplementary material for: Salivary Digestion Extends the Range of Sugar-Aversions in the German Cockroach
Source: Insects. 2021 Mar 21;12(3):263. doi: 10.3390/insects12030263 (PMC8003998; doi:10.3390/insects12030263)
Supplement: Supplementary file 1 [file insects-12-00263-s001.pdf]

Supplementary Information

for

Salivary Digestion Extends the Range of Sugar-Aversions in the German Cockroach

Ayako Wada-Katsumata* and Coby Schal*

[1] Department of Entomology and Plant Pathology and W.M. Keck Center for Behavioral Biology, North Carolina State University, Raleigh, North Carolina USA
* Correspondence: A.W-K., akatsum@ncsu.edu; C.S., coby@ncsu.edu

Table S1. Feeding responses to sugars in the acceptance-rejection assay, and sugar consumption

Different letters indicate significant differences between sugar treatments within each strain ($p < 0.05$).

Percentage response in wild-type females						
Concentration (mM)	Glucose (21)	Trehalose (21)	Sucrose (21)	Maltose (21)	Maltotriose (21)	Fructose (21)
0.1	0.0	0.0	0.0	0.0	0.0	0.0
1.0	0.0	9.5	9.5	28.6	38.1	4.8
10	9.5	61.9	42.9	76.2	85.7	23.8
100	52.4	90.5	90.5	100.0	100.0	66.7
1000	100.0	100.0	100.0	100.0	100.0	100.0
**Percentage response in glucose-averse females**						
Concentration (mM)	Glucose* (20)	Trehalose (31)	Sucrose (31)	Maltose (31)	Maltotriose (31)	Fructose (31)
0.1	100.0	0.0	0.0	0.0	0.0	0.0
1.0	65.0	12.9	16.1	25.8	38.7	3.2
10	25.0	48.4	41.9	67.7	67.7	29.0
100	0.0	90.3	83.9	93.5	100.0	64.5
1000	0.0	100.0	100.0	100.0	100.0	100.0

Sugar acceptance and rejection in wild-type and glucose-averse females, EC_{50} (mM, 95% CI)						
	Glucose	Trehalose	Sucrose	Maltose	Maltotriose	Fructose
Wild-type	76.20 (57.45, 94.95)	7.41 (5.20, 9.62)	11.58 (8.17, 15.00)	2.63 (1.83, 3.43)	1.63 (1.12, 2.14)	34.44 (24.18, 44.69)
Glucose-averse	2.22 (1.48, 2.96)	9.35 (6.44, 12.26)	11.51 (7.59, 15.44)	3.81 (2.48, 5.13)	2.29 (1.45, 3.13)	33.13 (23.15, 43.12)

Table S1 continued

Water and sugar consumption (nl), Mean ± SE (n)					
Solution (mM)	Wild-type		Glucose-averse		t-test ($p < 0.05$)
Water	81.6 ± 16.7 a	(26)	49.3 ± 18.3 a	(13)	t = -1.26, p = 0.217
					ANOVA, Tukey's HSD ($p < 0.05$)
Glucose 10	240.1 ± 47.3 bc	(21)	36.2 ± 14.7 c	(21)	F (5, 122) = 18.3, p < 0.0001
Glucose 100	733.6 ± 210.4 b	(21)	6.6 ± 4.5 c	(21)	
Glucose 1000	1719.5 ± 300.2 a	(23)	3.3 ± 3.3 c	(21)	
Trehalose 10	92.1 ± 25.4 b	(21)	223.7 ± 70.9 b	(21)	F (5, 120) = 15.9, p < 0.0001
Trehalose 100	1207.2 ± 218.9 b	(21)	345.4 ± 168.3 b	(21)	
Trehalose 1000	3009.9 ± 575.5 a	(21)	697.4 107.8 b	(21)	
Sucrose 10	108.6 ± 23.5 c	(21)	88.8 ± 39.1 c	(21)	F (5, 120) = 44.1, p < 0.0001
Sucrose 100	1072.4 ± 161.7 b	(21)	125.0 ± 30.1 c	(21)	
Sucrose 1000	4029.6 ± 490.9 a	(21)	523.0 ± 88.3 c	(21)	
Maltose 10	480.3 ± 70.0 b	(21)	141.4 ± 27.5 b	(21)	F (5, 120) = 18.7, p < 0.0001
Maltose 100	2023.0 ± 415.2 b	(21)	210.5 ± 58.0 b	(21)	
Maltose 1000	5486.8 ± 1051.2 a	(21)	513.2 ± 140.9 b	(21)	
Maltotriose 10	552.6 ± 102.1 c	(21)	68.5 ± 17.5 c	(25)	F (5, 132) = 29.9, p < 0.0001
Maltotriose 100	2319.1 ± 246.4 b	(21)	460.5 ± 80.3 c	(25)	
Maltotriose 1000	4332.2 ± 659.5 a	(21)	1140.4 ± 91.9 bb	(25)	
Fructose 10	151.3 ± 42.7 b	(21)	128.3 ± 33.9 b	(21)	F (5, 120) = 12.7, p < 0.0001
Fructose 100	500.0 ± 150.1 b	(21)	417.8 ± 92.0 b	(21)	
Fructose 1000	1434.2 ± 287.3 a	(21)	1309.2 ± 171.2 a	(21)	

Table S2. Effects of saliva on sugar degradation and feeding responses in the acceptance-rejection assay

Different letters indicate significant differences between treatments within each strain ($p < 0.05$).

% acceptance of sugars in wild-type (WT) and glucose-averse (GA) females					
Solution	Starved WT females		Starved GA females		Chi-square test
Water	100.0	(20)	100.0	(20)	
Water + Saliva of GA females	100.0	(20)	100.0	(20)	-
Water + Saliva of WT females	100.0	(20)	100.0	(20)	
Solution	Non-starved WT females		Non-starved GA females		
Water	0.0	(20)	0.0	(20)	
Water + Saliva of GA females	0.0	(20)	0.0	(20)	-
Water + Saliva of WT females	0.0	(20)	0.0	(20)	
Glucose + Water	80.0 a	(20)	0.0	(20)	
Glucose + Saliva of GA females	80.0 a	(20)	0.0	(20)	-
Glucose + Saliva of WT females	80.0 a	(20)	0.0	(20)	
Trehalose + Water	94.4 a	(18)	88.9 a	(18)	
Trehalose + Saliva of GA females	83.3 a	(18)	38.9 b	(18)	$X^2(5) = 28.46$, $p < 0.0001$
Trehalose + Saliva of WT females	83.3 a	(18)	38.9 b	(18)	
Sucrose + Water	85.0 a	(20)	86.4 a	(22)	
Sucrose + Saliva of GA females	80.0 a	(20)	40.9 b	(22)	$X^2(5) = 25.15$, $p = 0.0001$
Sucrose + Saliva of WT females	85.0 a	(20)	40.0 b	(20)	
Maltose + Water	95.5 a	(22)	90.9 a	(22)	
Maltose + Saliva of GA females	77.3 a	(22)	13.6 b	(22)	$X^2(5) = 64.22$, $p < 0.0001$
Maltose + Saliva of WT females	72.7 a	(22)	13.6 b	(22)	
Maltotriose + Water	100.0 a	(20)	100.0 a	(22)	
Maltotriose + Saliva of GA females	95.0 a	(20)	36.4 b	(22)	$X^2(5) = 59.59$, $p < 0.0001$
Maltotriose + Saliva of WT females	95.0 a	(20)	35.0 b	(20)	
Fructose + Water	86.4 a	(22)	86.4 a	(22)	
Fructose + Saliva of GA females	86.4 a	(22)	81.8 a	(22)	$X^2(5) = 0.31$, $p = 0.998$
Fructose + Saliva of WT females	86.4 a	(22)	86.4 a	(22)	

Table S3. Involvement of salivary glucosidases in sugar degradation

Different letters indicate significant differences between treatments ($p < 0.05$).

% acceptance for sugars in Glucose-averse females			
Solution	% of females		Chi-square test
Glucose	25.0 a	(20)	n.s
Glucose + Acarbose	25.0 a	(20)	
Glucose + Saliva	25.0 a	(20)	
Glucose + Saliva + Acarbose	25.0 a	(20)	
Maltose	96.0 a	(25)	$X^2(3) = 61.92$, $p < 0.0001$
Maltose + Acarbose	92.0 a	(25)	
Maltose + Saliva	8.0 b	(25)	
Maltose + Saliva + Acarbose	84.0 a	(25)	
Maltotriose	100.0 a	(25)	$X^2(3) = 52.94$, $p < 0.0001$
Maltotriose + Acarbose	100.0 a	(25)	
Maltotriose + Saliva	40.0 b	(25)	
Maltotriose + Saliva + Acarbose	100.0 a	(25)	
Trehalose	90.0 a	(20)	$X^2(3) = 21.51$, $p < 0.01$
Trehalose + Acarbose	85.5 a	(20)	
Trehalose + Saliva	25.0 b	(20)	
Trehalose + Saliva + Acarbose	25.0 b	(20)	
Sucrose	90.0 a	(20)	$X^2(3) = 31.85$, $p < 0.01$
Sucrose + Acarbose	90.0 a	(20)	
Sucrose + Saliva	40.0 b	(20)	
Sucrose + Saliva + Acarbose	90.0 a	(20)	
Fructose	85.0 a	(20)	n.s
Fructose + Acarbose	85.0 a	(20)	
Fructose + Saliva	85.0 a	(20)	
Fructose + Saliva + Acarbose	85.0 a	(20)	

Table S4. Salivary proteins and alpha-glucosidase activity in cockroach saliva

Different letters indicate significant differences among strains and sexes ($p < 0.05$).

	Alpha-glucosidase activity using p-Nitrophenol (Mean ± SE mU/µl saliva)			
	WT female (5)	GA female (5)	WT male (5)	GA male (5)
Saliva	129.0 ± 11.3 a	69.6 ± 11.2 b	88.2 ± 4.7 b	56.2 ± 3.1 b
Saliva + acarbose	24.2 ± 2.6 c	22.7 ± 2.6 c	32.3 ± 1.4 c	25.0 ± 0.8 c
ANOVA，Tukey HSD ($p < 0.05$)	$F (7, 36) = 46.5$, $p < 0.0001$			

	Total protein (Mean ± SE ng/µl saliva)			
	WT female (6)	GA female (8)	WT male (6)	GA male (7)
Saliva	245.4 ± 19.4 a	114.1 ± 10.0.6 b	138.6 ± 8.5 b	126.7 ± 12.5 b
ANOVA，Tukey HSD ($p < 0.05$)	$F (3, 23) = 20.3$, $p < 0.0001$			
